# Driving Electron Transfer in Photosystem I Using Far-Red Light: Overall Perspectives

**DOI:** 10.3390/plants14213384

**Published:** 2025-11-05

**Authors:** Jimit Patel, Amen ElMasadef, Abraham Peele Karlapudi, Katayoun Etemadi, K. V. Lakshmi, Art van der Est, Divya Kaur

**Affiliations:** 1 Department of Chemistry, Brock University, St. Catharines, ON L2S 3A1, Canada; jp19fk@brocku.ca; 2 Department of Biological Sciences and Biotechnology, Brock University, St. Catharines, ON L2S 3A1, Canada; ae19ry@brocku.ca (A.E.); ke22ou@brocku.ca (K.E.); avanderest@brocku.ca (A.v.d.E.); 3 Department of Biotechnology, Vignan’s Foundation for Science, Technology and Research, Guntur 522213, Andhra Pradesh, India; abrahampeelekarlapudi@gmail.com; 4 Department of Chemistry and Chemical Biology, The Baruch ’60 Center for Biochemical Solar Energy Research, Rensselaer Polytechnic Institute, Troy, NY 12180, USA; k.lakshmi04@gmail.com

**Keywords:** far-red photosynthesis, Photosystem I, reaction center, electron transfer reaction, far-red light photoacclimation, FaRLiP

## Abstract

Photosystem I (PSI) is a photosynthetic protein–pigment complex that, upon photoexcitation, transfers electrons to ferredoxin, facilitating the production of NADPH. Isolated PSI reaction centers (RCs) have also been used in hybrid systems to reduce protons and produce ‘biohydrogen’. This review article examines how various cyanobacteria with similar photosynthetic machinery utilize different wavelengths of light to execute photosynthetic electron transport through PSI. Key factors, such as, the structure of the electron transfer cofactors, the protein environment surrounding the primary donor pigments and hydrogen-bonding interactions with the surrounding protein matrix are analyzed to understand their roles in maintaining efficient electron transfer when it is driven using photons of different energies. We compare PSI complexes with known atomic structures from four species of cyanobacteria, *Thermosynechococcus elongatus*, *Acaryochloris marina*, *Halomicronema hongdechloris*, and *Fischerella thermalis*. *T. elongatus* is typical of most oxygenic photosynthetic organisms in that it requires visible light and uses only chlorophyll *a* (Chl *a*) in PSI. In contrast, *H. hongdechloris* and *F. thermalis* are photoacclimating species capable of producing Chl *f* and Chl *d* that use red light when little visible light is available. *A. marina*, on the other hand, is adapted to red light conditions and consistently utilizes Chl *d* as its primary photosynthetic pigment, maintaining a stable pigment composition. Here, we explore the structural and functional differences between the PSI RCs of these organisms and the impact of these differences on electron transport. The structural differences in the cofactors influence both the absorption wavelengths of the cofactors and the energy levels of the intermediate states of electron transfer. An analysis of the surrounding protein shows how it has been adapted and underscores the interplay between the pigment structure, protein environment, and hydrogen bonding networks in tuning the efficiency and adaptability of photosynthetic mechanisms across different species of cyanobacteria.

## 1. Introduction

Efficient utilization of incident light is crucial for the survival of photosynthetic organisms, which have evolved various strategies to adapt to changes in the wavelength and intensity of light. In this review, we discuss the far-red light acclimation and adaptation strategies that have resulted in different forms of PSI from distinct species of cyanobacteria. However, prior to delving into the details, we present an overview of the light reactions and electron transport cofactors, highlighting recent advances in the field.

### 1.1. Oxygenic Photosynthesis

Oxygenic photosynthesis is the principal mechanism by which sunlight is converted into chemical energy, making it one of the most fundamental biological processes [1,2,3]. Cyanobacteria or their early ancestors were the first organisms to perform oxygenic photosynthesis, using water as an electron donor and releasing dioxygen. While dioxygen is key to sustaining aerobic life on the Earth, its production by early oxygenic photosynthetic organisms led to what is known as the Great Oxygenation Event that nearly caused the extinction of all life [1,4,5].

In oxygenic photosynthesis, two distinct photosystems, Photosystem II (PSII) and Photosystem I (PSI) [6], perform light-induced electron transfer to convert light energy into chemical energy. The oxidizing and reducing species generated by electron transfer are used to produce NADPH and dioxygen. The proton transfer that accompanies the electron transfer is used to drive the synthesis of ATP [6,7,8,9]. The reducing equivalents stored as NADPH and ATP are subsequently used for CO_2_ fixation in the dark reactions [2]. The evolution of photosynthesis has been a complex process that has unfolded over billions of years from primitive anoxygenic bacteria to oxygenic cyanobacteria, algae, and, ultimately, higher plants [10,11,12,13]. In eukaryotic organisms, the photosynthetic apparatus is housed in chloroplast organelles resulting from an endosymbiotic relationship between a cyanobacterium and a non-photosynthetic microorganism [14].

### 1.2. Far-Red Light Photosynthesis

One of the main challenges facing photosynthetic organisms is responding to the changes in the light conditions. The wavelengths and intensity of the incident light vary in different environments and is subject to rapidly change. Different adaptation strategies allow these organisms to deal with these challenges. For example, green sulfur bacteria employ chlorosomes with exceptionally high absorption cross-sections as antenna that allow the organism to survive in extremely low-light environments [11]. Purple bacteria employ bacteriochlorophylls that absorb photons in the IR region beyond 800 nm, allowing them to live in the deep anaerobic layers of lakes or microbial mats with little visible light [15]. Oxygenic photosynthetic organisms have evolved mechanisms that primarily deal with changing light intensity. Cyanobacteria, for example, can undergo state transitions in which the flow of excitation energy from the phycobilisome antenna to the photosystems is altered under different light conditions [16]. These organisms can also alter the ratio of Chl *a*, which absorbs mostly violet and orange light, to Chl *b*, which absorbs mostly blue and yellow light, to adapt their absorption profiles. For many years, it was thought that oxygenic photosynthesis required the absorption of photons in the visible region to provide sufficient energy to drive water oxidation. However, the discovery of the cyanobacteria *A. marina* and *H. Hongdechloris* containing Chl *d* and Chl *f*, respectively, demonstrated that cyanobacteria can also thrive under far-red light conditions [17,18].

Recent advances have provided insight into the molecular diversity and evolutionary pathways of far-red light photosynthetic organisms [19]. Advanced crystallographic and biochemical analyses reveal the details of the mechanisms underlying the red shift of the photosynthetic pigments. For example, a recent study demonstrated that in allophycocyanins, the light-harvesting proteins found in cyanobacteria, are red-shifted as a result of altered protein–cofactor interactions that lead to a conformational change in the phycocyanobilin chromophore [20,21]. Such knowledge has the potential to allow for the engineering of photosynthetic organisms that utilize a broader spectrum of light, thus improving both efficiency and adaptability and paving the way for innovations in agriculture and biotechnology [20,22].

**Figure 1:**
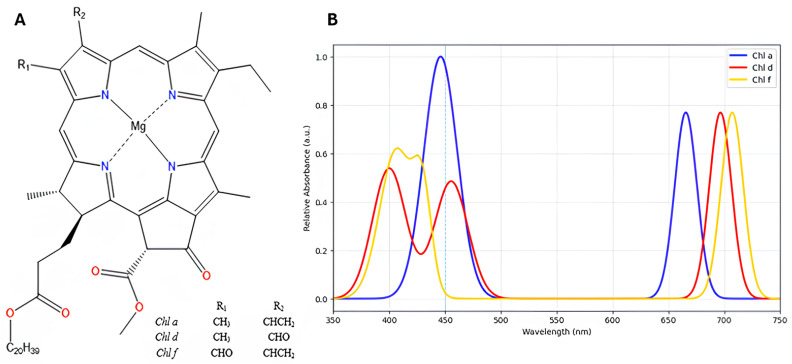
(**A**) Chemical structures of chlorophyll *a*, *d*, and *f*. (**B**) Absorbance spectra generated using the experimental extinction coefficient values of chlorophyll *a*, *d* and *f* in 100 % methanol, as reported in Table 2 of Li et al. (2012) [29]. The experimental spectrum and λ_max_ values can be found in Figure 1 and Table 2, respectively, of the reference Li et al. (2012) [29].

The term far-red photosynthesis is used to refer to the use of light in the far-red infrared region (700–800 nm) [23] in oxygenic organisms [23,24]. Cyanobacteria, which normally only use the visible part of the spectrum, can perform far-red photosynthesis through two related mechanisms involving Chl molecules that differ in their substituent groups [25]. The first is the adaptation of certain strains of cyanobacteria to niche environments where the incident light is consistently in the far-red region. In this case, the organisms synthesize long wavelength absorbing chlorophyll molecules, such as, Chl *d*, and PSI and PSII adapt to specifically bind these cofactors. The second is a dynamic process in which the organism responds to changes in the light conditions, upregulating the synthesis of modified chlorophylls, such as, Chl *d* and Chl *f* and modified versions of the photosynthetic proteins to bind the pigments when needed through a process known as far-red light photoacclimation (FaRLiP) [23,26,27,28] (Figure 1).

The acclimation and adaptation of photosynthetic organisms to FRL has been reviewed extensively in the literature [17,30,31,32,33]. These reviews are focused on the sensing and response to FRL and the evolution of these mechanisms. However, the use of FRL photons has consequences for the energetics of electron transfer in the RCs. With the availability of the near-atomic resolution structures of the RCs, it is now possible to investigate the adaptation of the electron transfer cofactors and the respective binding sites in greater detail. Here, we review the structure of PSI isolated from red-shifted cyanobacterial species and the impact of far-red absorbing Chl molecules on the electron transport chain. We begin with a brief overview of the structure of the electron transport chain of PSI from the Chl *a*-containing cyanobacterium *T. elongatus*.

### 1.3. Structure and Electron Energy Transfer in Far-Red-Light-Adapted Photosystem I

**Figure 2:**
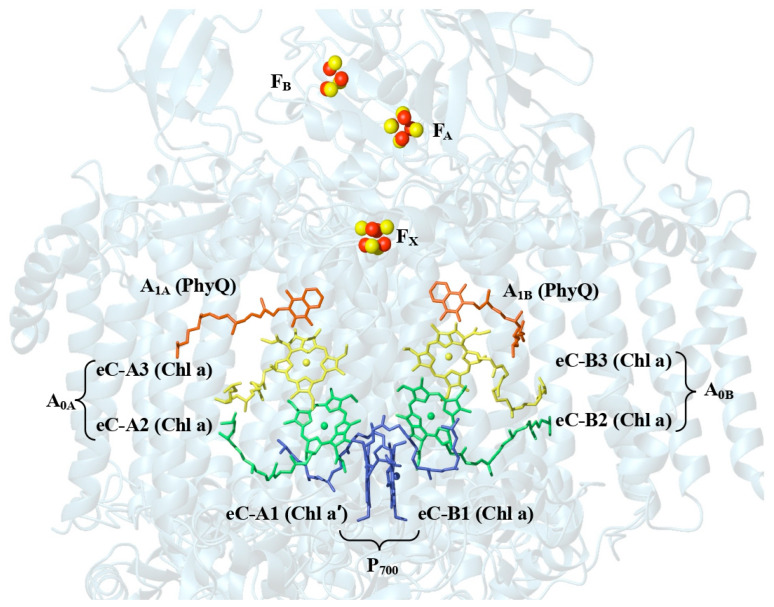
Arrangement of the cofactors that participate in the electron transfer chain of *T. elongatus* (PDB. ID: 1JB0) [9].

PSI from *T. elongatus* contains 12 protein subunits that bind to 96 Chl *a* molecules, 22 carotenoids, 3 four-iron–four-sulfur [4Fe4S] clusters, and two phylloquinone molecules [5]. The core of the RC is a protein heterodimer comprising the membrane-spanning polypeptides PsaA and PsaB that house most of the electron transport cofactors (Figure 2). The pair of chlorophyll molecules labeled eC-A1 and eC-B1 that are shown in blue near the lumenal side of the RC act as the primary electron donor P_700_. eC-B1 is Chl *a* molecule, while eC-A1 is Chl *a*′, the C13^2^-epimer of Chl *a*. Two nearly symmetrical branches of cofactors extend across the membrane from P_700_, each containing two Chl *a* molecules (shown in green and blue in Figure 2) and a phylloquinone molecule (shown orange). The A- and B-branch of cofactors converge at the [4Fe4S] cluster F_X_ and the terminal [4Fe4S] clusters F_A_ and F_B_ are bound to the PsaC subunit on the stromal side of the complex.

Photoexcitation of the primary donor results in electron transfer from P_700_ to F_X_ in both the A- and B-branch [34]. A variety of models have been proposed for the initial steps of this process [35,36]. However, there is general agreement that the charge-separated state P_700_^+^A_0A_^−^ or P_700_^+^A_0B_^−^ is formed within a few picoseconds in either the A- or B-branch. In photo-accumulation experiments, it has been shown that the transferred electron is distributed over two Chl *a* molecules. Thus, we refer to the pair of Chl *a* molecules eC-2 and eC-3 in each branch as the primary acceptor A_0_ [7]. Subsequent electron transfer to the phylloquinone acceptors A_1A_ and A_1B_ [37] and the three [4Fe-4S] clusters F_X_, F_A_, and F_B_ stabilizes the initial charge separation providing sufficient time for the diffusion limited reduction of P_700_^+^ by soluble plastocyanin and the transfer of the electron from the reduced (F_A_/F_B_)^−^ clusters to soluble ferredoxin or flavodoxin [38,39]. The distance between the cofactors and their redox potentials are finely tuned to optimize the electron transfer resulting in a quantum yield of near unity. The efficient production of the reductant NADPH is used to drive CO_2_ fixation [38]. It is known from a large body of site-directed mutagenesis and quinone exchange studies that even small changes in the cofactors or the protein environment typically lead to impairment of function [40,41]. Thus, it is of interest to understand how PSI RCs from organisms using FRL maintain highly efficient electron transfer processes.

## 2. Chl *d* Containing PSI

In the marine cyanobacterium *A. marina*, red light-absorbing Chl *d* accounts for 91–97% of the chlorophyll [39,42]. The remaining 3–9% is Chl *a* [43]. *A. marina* was discovered in 1996 when it was extracted from *Lissoclinum patella*, a colonial ascidian collected from the coast of Palau islands in 1993 [16]. Since then, many other strains of *Acaryochloris* have been discovered to use far-red light to drive the photosynthetic reactions [44]. These strains differ in the specific wavelengths of far-red light used, which has enabled adaptation to diverse ecological environments [45]. While most of them are known to utilize far-red light, *A. marina* MBIC11017 has been shown to acclimate to white light by altering the ratio of PSI:PSII and increasing the number of phycobilisomes [46]. In PSI from *A. marina*, the absorption maximum of the primary donor P_740_ is red-shifted to 740 nm as a result of the presence of Chl *d* [46]. This change raises intriguing questions about its effect on the energetics of electron transport and the structure–function relationships. The recent cryo-EM structure of PSI from *A. marina* with a resolution of 2.58 Å published by Hamaguchi et al. [41]. facilitates detailed comparisons of the electron transfer cofactors of *T. elongatus* and *A. marina*.

### 2.1. Electron Transport Chain (ETC) of Acaryochloris marina PSI

In *T. elongatus*, P_700_ is a Chl *a*/Chl *a*′ heterodimer and A_0_ is a Chl *a* homodimer. In contrast, P_740_ is a Chl *d*/Chl *d*′ heterodimer [47,48], and the eC-2 and eC-3 cofactors in each branch are a Chl *d* and pheophytin, respectively, in *A. marina*. The phylloquinones, A_1A_ and A_1B_, and three [4Fe4S] clusters F_X_, F_A_, and F_B_ are similar in both organisms. Despite the differences in some of the cofactors, the arrangement of these pigments is identical in *A. marina* and *T. elongatus* [39].

### 2.2. Absorption Characteristics of P_740_

The change in the vinyl group substituent at the C3 position of Chl *a* to a formyl group in Chl *d* (Figure 1) causes a red shift of 25 nm in the Qy absorption band measured in acetone [49]. Qualitatively, this effect can be attributed to the stabilization of the lowest unoccupied molecular orbital (LUMO) due to greater delocalization onto the more electronegative formyl group [50]. In the protein environment, the Qy absorption maxima of P_700_ and P_740_ are both red-shifted compared to the respective Chls in organic solvents as a result of the excitonic interactions between the chlorophylls of the respective dimers and protein cofactor interactions. The Qy maximum for P_700_ is shifted by ~800 cm^−1^ compared to Chl *a* in solution, whereas the shift for P_740_ relative to Chl *d* in vitro is ~1000 cm^−1^. Thus, although the different substituent groups of the Chl are responsible for the red shift of P_740_, a difference in the interaction between the two Chls in the dimer and/or a difference in the influence in the protein environment also appears to play a role. The metal coordination properties of the central Mg^2+^ ion of Chl *a* and Chl *d* are similar [51], and the nature and arrangement of the axial His ligand of P_740_ and P_700_ are essentially the same in the respective X-ray crystal structures. This suggest that the change in vinyl to formyl at the C3 position does not affect the binding to protein [25,47]. Nonetheless, protein-cofactor interactions are expected play an important role in tuning the electronic properties of P_740_.

### 2.3. The Midpoint Potential and Electronic Asymmetry of P_740_

The midpoint potential (*E_m_*) of the primary donor P_740_ has been estimated as +425–+450 mV [48,52,53] vs. SHE. This is well within the +400–+470 mV range for the *E_m_* of P_700_ in Chl *a*-containing cyanobacteria [54]. In contrast, the midpoint potential of Chl *a* and Chl *d* in vitro in acetonitrile are +810 mV and +880 mV, respectively, [55]. The lower *E_m_* values of P_740_ and P_700_ in vivo can be ascribed to greater stabilization of the oxidized dimer in the protein environment compared to the corresponding oxidized monomers in solution. The protein sequence and structure in the vicinity of P_740_ and P_700_ are highly conserved in *A. marina* and *T. elongates*, suggesting similar stabilization in vivo. Within the uncertainty of the reported midpoint potentials, it appears that the higher oxidation potential of Chl *d* compared to Chl *a* (~70 mV) observed in solution is compensated for by subtle differences in the chlorophyll and/or chlorophyll–protein interactions in P_700_ and P_740_. It is not surprising that these interactions result in similar *E_m_* of P_740_^+^ and P_700_^+^, since both are re-reduced by the same soluble electron donors, plastocyanin or cytochrome *f*.

**Figure 3:**
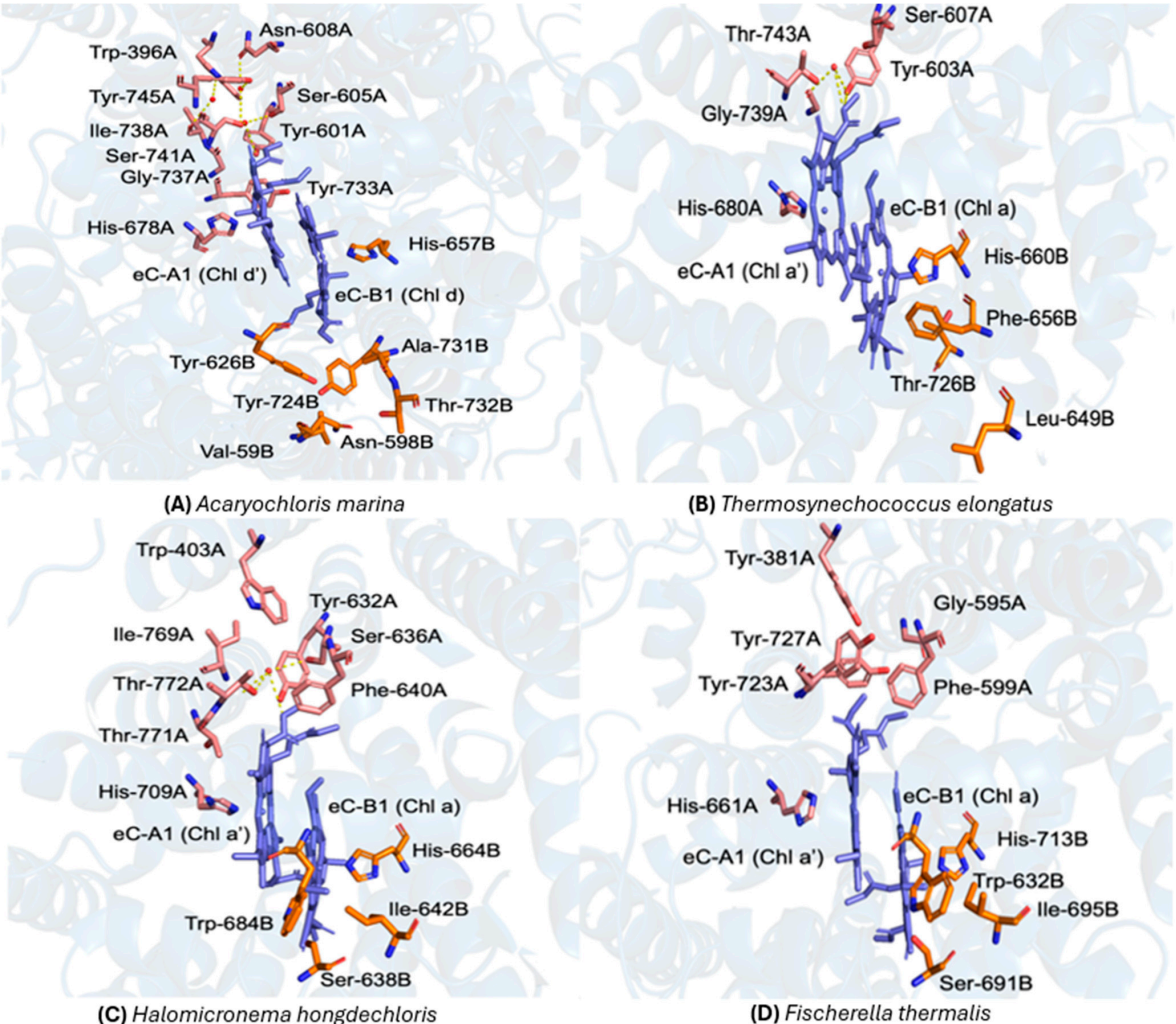
Comparison of the primary donor sites and their local protein environments in Photosystem I (PSI) from four cyanobacterial species. (**A**) *Acaryochloris marina* (P740; PDB ID: 7COY) [39]: the Chl *d*′ (eC-A1) and Chl *d* (eC-B1) molecules are shown in blue, with surrounding residues from P_A_ and P_B_ depicted in pink and orange, respectively, and this color scheme is applied to all organisms in this study. Key A-branch residues include Trp-396A, Ile-738A, Asn-608A, Tyr-601A, Ser-605A, Tyr-733A, Tyr-745A, Ser-741A, and Gly-737A, coordinated by His-678A; B-branch residues include Tyr-724B, Ala-731B, Thr-732B, Val-594B, Asn-598B, and Tyr-626B, coordinated by His-657B. Three nearby water molecules (red spheres) and their putative hydrogen bonds (yellow dashed lines) are indicated [39]. (**B**) *Thermosynechococcus elongatus* (P700; PDB ID: 1JB0) [6]: Chl *a*′ (eC-A1) and Chl *a* (eC-B1) are shown in blue. The A-branch includes Tyr-603A, Ser-607A, Gly-739A, Thr-743A, and His-680A, while the B-branch comprises Phe-656B, Leu-649B, Thr-726B, and His-660B. A water molecule (red sphere) and its hydrogen-bond network (yellow dashed lines) illustrate stabilizing interactions near the donor [6]. (**C**) *Halomicronema hongdechloris* (P700; PDB ID: 6KMX) [56]: Chl *a*′ (eC-A1) and Chl *a* (eC-B1) are shown in blue. The water molecule is shown as a red sphere. (**D**) *Fischerella thermalis* (PDB ID: 7LX0): the primary donor environment closely resembles that of *H. hongdechloris* [57]. At the reported 2.96 Å resolution, only strongly bound waters could be modeled; thus, the apparent absence of water or hydrogen-bond interactions likely reflects limited structural resolution rather than a true lack of water molecules [58].

The structure of P_740_ in PSI from the *A. marina* and the surrounding protein environment is shown in Figure 3A. Here, eC-A1 is a Chl *d*′ molecule and the eC-B1 is Chl *d* and there are three water molecules in the vicinity of eC-B1. The water molecules form putative hydrogen bonds with the neighboring amino acid residues Tyr601A, Ser605A, Asn608A, Ser741A, Tyr745A, and Chl *d*′ [39]. In contrast, PSI from *T. elongatus* contains just one water molecule in the vicinity of P_700_, that possibly forms hydrogen bonds with Tyr603A, Ser607A, Thr743A, and Chl *a*′ (Figure 3B) [6].

The differences in the hydrogen (H) bonding network in the vicinity of P_700_ and P_740_ may be responsible for maintaining the midpoint potential and is expected to influence the asymmetry of the charge distribution. In *C. reinhardtii*, site-directed mutagenesis studies have shown that altering the axial His ligand to eC-B1 changes the midpoint potential of P_700_ and the unpaired electron spin density distribution of the P_700_^+^ radical cation [59]. Corresponding changes to the axial His ligand of eC-A1 had no effect. Thus, it was concluded that the charge and unpaired electron spin density distribution on P_700_^+^ is primarily localized on the eC-B1 chlorophyll. Since H-bonds have an electron withdrawing effect, it is expected that the asymmetric H-bonding to P_700_^+^ would draw electron density towards the Chl with more and/or stronger H-bonding interactions, leading to the localization of positive charge density on the other Chl [60,61]. Indeed, it has been shown in purple bacterial reaction centers that there is a linear correlation between the midpoint potential and the total enthalpy of the hydrogen (H)-bonds to the carbonyl groups of the primary donor chlorophylls [60]. Thus, the larger number of water molecules and H-bonding interactions in the vicinity of eC-A1 in *A. marina* might be expected to result in a higher midpoint potential and higher electron hole localization on eC-B1 in P_740_^+^ compared to P_700_^+^. Based on a computational study combining electrostatic continuum calculations and QM/MM methods by Saito et al. (2011) [62], the *Em* of Chl *a* and Chl *a*′ of P_700_ suggested an eC-A1^•+^: eC-B1^•+^ ratio of 28:72 due to the strong putative H-bond between P_A_ and Thr743A as one of the main factors. A study of the molecular geometry and vibrational frequency using DFT methods employing a B3LYP functional and a6-31G(d) basis set with a PCM solvent model calculated the eC-A1^•+^:eC-B1^•+^ ratio of P_740_ as 60:40, without specifying the branching ratio [63]. However, computational and electron nuclear double resonance (ENDOR) studies show that the electron spin density distribution of P_740_^+^+ and P_700_^+^ are similar, which suggests that the difference in the number of water molecules and the H-bonding interactions have a relatively minor effect [64]. More research is needed to better understand the underlying reasons for the asymmetric electron transfer and asymmetry of spin and charge distribution in the two organisms.

### 2.4. Electron Transfer Energetics and the Protein Environment

The local protein environment surrounding the Chls that form the dimeric primary acceptor A_0_ and the secondary phylloquinone acceptor A_1_ is also conserved in PSI from the different species of cyanobacteria, except for the axial ligands of eC-3. In PSI from *T. elongates*, eC-3A and eC-3B are ligated by Met688A and Met668B, respectively. However, eC-3A and eC-3B are pheophytin molecules in *A. marina* PSI lacking a central Mg(II) ion and therefore do not contain axial ligands [65]. The Met residue in the A-branch coordinated to eC-3A in *T. elongatus* is conserved *A. marina* PSI; however, the corresponding residue in the B-branch of *A. marina* is a Leu. This difference does not appear to significantly impact the rate of electron transfer in the two branches in *A. marina*. This is in stark contrast to PSI from *T. elongatus* and *C. reinhardtii*, where mutation of Met688A and Met668B profoundly affects the electron transfer rates [66,67,68]. A recent study of the initial charge separation kinetics by Petrova et al. [65] suggests that the use of Pheo as the A_0_ acceptor in *A. marina* is necessary to provide the driving force required for forward electron transfer from P_740_. The difference in the absorption wavelength suggests that the excited state P_740_* is ~100 meV lower in energy than P_700_* relative to the respective ground states. Therefore, this loss in excitation energy must be compensated for by lowering the energy of the charge-separated state. Since the midpoint potential of P_700_ and P_740_ are similar, the energy of the charge-separated state can only be lowered by rendering the midpoint potential of the primary acceptor more positive. Ultrafast optical spectroscopy has suggested that the midpoint potential of Pheo in *A. marina* is ~100 mV more positive than A_0_ in *T. elongatus*. However, the uncertainty in this estimation is fairly large because the values obtained depend on the assumed mechanism of the initial charge separation and stabilization used in the analysis. A recent study by Noji et al. [69] addressed this issue by solving the linear Poisson–Boltzmann equation to estimate the midpoint potential for the electron transfer cofactors. They found that the midpoint potential of eC-2A is ~200 mV more positive in *A. marina* than in *T. elongatus*. However, if the calculation is repeated using the *A. marina* structure with Chl *a* in the binding site, the midpoint potential is essentially the same as that in *T. elongatus*. Hence, it appeared that the difference in the midpoint potential is inherent to the chemical structure of the Chl and does not arise from differences in the protein environment. Similarly, the midpoint potential of eC-3 (Pheo-A) in *A. marina* is ~200 mV more positive than eC-3 (Chl *a*) in *T. elongatus*. Thus, the change from Chl *a* in *T. elongatus* to Pheo-A in *A. marina* keeps eC-3^−^ energetically lower than the eC-2^−^.

The secondary phylloquinone acceptors, A_1A_ and A_1B,_ are similar in *A. marina* and *T. elongatus*. In both organisms, the quinones are involved in hydrophobic interactions with a tryptophan and Leu residue and form a single putative hydrogen bond to a backbone NH group. In *T. elongatus*, the H-bonding partners are Leu722A and Leu706B, respectively, [6]. In other cyanobacteria, such as, *Gloeobacter*, the putative hydrogen bond in the B-branch is a Met [70]. This pattern is reversed in *A. marina*, where A_1A_ forms a putative hydrogen bond to Met720A while A_1B_ to Leu665B.

The conformation of the phytyl tail of the phylloquinone in the A_1B_ site differs in *A. marina* and *T. elongatus* [39,42]. Shen and coworkers speculate that this may arise from structural changes as Met acts as the axial ligand to A_0_ in *T. elongatus* and Leu in *A. marina* [42]. Not surprisingly, the terminal [4Fe4S] clusters in *A. marina* are nearly identical to those in *T. elongatus*, indicating that the midpoint potentials and electron transfer properties are likely unaffected by the other changes in the electron transfer chain [39].

## 3. Far-Red Light Photoacclimation (FaRLiP)

The second class of cyanobacteria that use FRL acclimate to longer wavelengths in a process known as FaRLiP, resulting in the incorporation of Chl *f* [22]. This type of Chl was first identified in the methanol extracts of stromatolites from Shark Bay, Western Australia and its presence led to the discovery of the cyanobacterium *H. hongdechloris*, a FaRLiP species that can sense light conditions and synthesize Chl *f* when needed [18,43,71,72,73]. The ability of these organisms to adapt to FRL was first discovered by Bryant and coworkers in the filamentous, non-heterocystous cyanobacterium *Leptolyngbya* sp. JSC-1 isolated from microbial mats in hot spring pools [74]. They found that this species acclimates dynamically to far-red light by remodeling PSI, PSII and phycobilisome proteins and their cofactors [27,30,31,75,76]. These alterations are associated with a cluster of genes that has now been identified in up to twenty species of cyanobacteria [31,32]. This gene cluster is highly conserved and consists of approximately twenty genes that encode for paralogs of the subunits of PSI, PSII, and phycobillisomes as well as the enzymes required for Chl *f* and Chl *d* biosynthesis [32,76]. The absorption of FRL by the phytochrome RfpA is thought to initiate a series of steps that lead to the activation of the regulator/transcription activator RfpB. The activated form of RfpB then activates the transcription of these genes to produce components of the photosynthetic apparatus optimized for FRL [77].

### 3.1. Characterization of Chl f-Containing Photosystems

The Chl *f* content in *H. hongdechloris* and *F. thermalis* varies depending on the wavelengths of the light under which the cultures are grown. Under white light, Chl *f* is minimal, but under FRL, it reaches 8–12.5% of the total amount of Chls [26]. In addition to activating the biosynthesis of Chl *f*, other photosynthetic pigments and proteins, such as the PSI subunits, are also altered [73]. In the modified form of PSI from *F. thermalis* grown under FRL, 6 out of 12 subunits (PsaA1, PsaB1, PsaL1, PsaI1, PsaF1, and PsaJ1) are replaced by alternative versions (PsaA2, PsaB2, PsaL2, PsaI2, PsaF2, and PsaJ2) that are encoded within the FaRLiP gene cluster [18,19,73,78]. The cryo-em 2.96 Å and cryo-em 3.19 Å structure of PSI from *F. thermalis* and cryo-em 2.41 Å *H. hongdechloris* grown under FRL conditions has shown that the locations of the Chls remain unchanged under FRL. However, several Chl binding sites observed in PSI from *T. elongatus*, and *A. marina* are absent in PSI from FRL *F. thermalis*, which results in the incorporation of Chl *f* as the major pigment. Based on the total Chl *f* content, ~7–8 Chl *f* molecules are expected to be found in PSI from FRL-acclimated *H. hongdechloris* and *F. thermalis* [28]. An important question is whether the Chl *f* in the FRL-acclimated species are involved in electron transfer. Nürnberg et al. (2018) initially reported biophysical studies of RCs from a FRL adapted cyanobacterium [19,75]. Based on the action spectra, it was proposed that charge separation in both PSI and PSII in *Chroococcidiopsis thermalis* PCC 7203 involves Chl *f*. Since the P_700_ absorbance was not red-shifted and the high midpoint potential of Chl *f* makes it a poor choice for A_0_, it was suggested that the accessory Chl site was the location for Chl *f*. Hastings and coworkers (2019) studied PSI from FRL-acclimated *F. thermalis* using FTIR difference spectroscopy, suggesting that P_700_ is a Chl *a*/Chl *a*′ dimer, and provided evidence of Chl *f* in the eC-2 sites [43,72,79]. However, time-resolved optical data spectroscopy indicates that the time scale on which the secondary charge separated states are formed following Chl *f* excitation is inconsistent with their participation in the electron transfer chain [80].

Determining the locations of Chl *f* in PSI from *H. hongdechloris* and *F. thermalis* is challenging as the resolution of the cryo-EM density maps is not sufficient to unequivocally distinguish between the formyl group of Chl *f* and the corresponding methyl group of Chl *a* [28]. Moreover, it is possible that a fraction of the sites in PSI from *F. thermalis* contain Chl *f* while the remainder are occupied by Chl *a*. Nonetheless, in PSI from *H. hongdechloris* and *F. thermalis* seven of the 96 and 89 Chls, respectively, were modeled as Chl *f*, all of which were located in the antenna [22,26,81]. In *H. hongdechloris*, six of the seven Chl *f* molecules were assigned to a network on the PsaA2 side of the complex near the interface with one of the two neighboring PSI complexes in the trimer. In *F. thermalis*, the lower resolution made the assignment more challenging and four possible binding sites were tentatively identified in the antenna. Most of the changes in the protein structure were due to alterations in amino acid side chains that allowed space for or provided putative hydrogen bond donors to the formyl oxygen on the A ring of Chl *f*. Additionally, several loop insertions and deletions were found in both structures. The assignment of Chl *f* only to antenna binding sites contradicted the earlier spectroscopic evidence suggesting that one or both of the accessory Chls of the electron transfer chain may be Chl *f* [79,82,83]. However, a rigorous analysis of the electron density maps taking into account the local energy landscape, the presence of possible hydrogen bond donors, and the nature of the axial ligand to the Chls has provided evidence for the locations of Chl *f* in the antenna [84].

### 3.2. The Electron Transfer Chain in FRL-PSI from H. hongdechloris and F. thermalis

The electron transfer cofactors in *H. hongdechloris* and *F. thermalis* are similar to those of *T. elongatus*, including the pseudo-C_2_ symmetric arrangement of the cofactors in the A- and B-branch, meaning that the electron transfer cofactors in the A- and B-branches are arranged with near-two-fold rotational symmetry around an axis normal to the membrane plane. This rotational symmetry is not exact because the protein environments in the two branches differ and there are slight differences in the orientations of the cofactors. The primary donor Chls eC-A1 and eC-B1 are directly ligated by His709A and His664B, respectively, in *H. hongdechloris* PSI (Figure 3C).

As is the case in PSI from *T. elongatus*, only one water molecule is present in the vicinity of P_A_ in PSI from FRL-acclimated *H. hongdechloris*, which forms putative hydrogen bonds with the Ser636A, Tyr632A, Gly768A, Thr772A, and methoxy O-13 group of P_A_ [26]. The P_B_ Chl of *H. hongdechloris* does not participate in hydrogen bonding [85]. The amino acids participating in hydrogen bonding around Chl *a*′ are also conserved in PSI from *H*. *hongdechloris* and *T. elongatus*. Thus, the energetics of electron transfer appears to be unaltered in FRL-acclimated PSI.

Another factor that can influence the energetics is the orientation of the pigments [7,86,87]. The fact that the orientation of eC-A1 and eC-B1 in P_700_ from *H. hongdechloris* and *T. elongatus* are virtually identical suggests that there is no significant difference in the *E_m_* and energetics of electron transfer. The excited state from which electron transfer is initiated is higher in energy than the available photons under FRL conditions, which suggests that electron transfer must be coupled to uphill energy transfer from Chl *f* on the periphery of the complex to P_700_ in the core [25].

### 3.3. Excitation Energy Transfer

The apparent lack of any change in the energetics in the electron transfer in PSI from FRL-acclimated *H. hongdechloris* and *T. thermalis* raises the intriguing question of how photons with less energy than that required for the photoexcitation P_700_ can initiate electron transfer. The input required for energy transfer from Chl *f* at 810 nm to Chl *a*, is ~770 cm^−1^ and well above thermal energy at ambient temperature. This implies that the energy transfer is coupled to low-frequency vibrational modes and since the Boltzmann factor describing the probability of excitation of such modes is 0.025, the uphill energy transfer is expected to be slow. Indeed, there is evidence that the energy transfer in photosystems containing red-shifted Chls is a two-step process in which the excitation is initially trapped on the red-shifted Chl, followed by slower uphill energy transfer [88]. Nonetheless, the rates of energy transfer are faster than expected and mathematical modeling suggests that the uphill energy transfer is entropy-driven. Because the ratio of Chl *f*/Chl *a* in the antenna is small, there are a large number of possible pathways for energy transfer from Chl *f* to Chl *a* [22]. Since the number of pathways is related to the probability of energy transfer, a small Chl *f*: Chl *a* ratio has been proposed to partially compensate for the intrinsically low probability of uphill transfer [89].

### 3.4. Comparative Analysis of the Protein Environment Surrounding P_700_ in H. hongdechloris, F. thermalis and T. elongatus

A recent comparative analysis of the protein environment of PSI from *F. thermalis* and *H. hongdechloris* revealed a high degree of structural similarity. However, a key distinction is in the absence of water molecules in PSI from *F. thermalis*, as observed in the cryo-EM 2.96 Å and 3.19 Å structure (PDB ID: 6PNJ, [90] 7LX0) [57]. While this observation pertains to the PSI core, it is important to note that Chl *f* molecules are localized at the periphery of the antenna, spatially distant from the electron transfer chain [22,28]. This raises the question of whether differences in the hydration state in the PsaA and PsaB subunits plays a functional role in FRL acclimation. Structural comparisons between *F. thermalis* and *H. hongdechloris* indicate only minor variations in the orientations of the amino acid side chains near the core, typically within 1 Å. Thus, while the absence of water molecules in the core is a noteworthy structural feature, its direct impact on FRL-driven electron transfer remains uncertain, especially given the peripheral location of Chl *f*.

**Figure 4:**
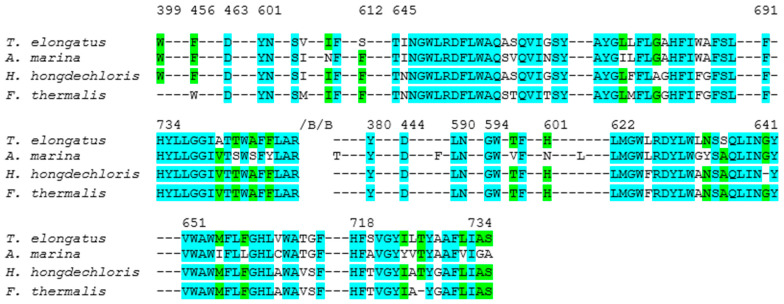
Alignment of PsaA sequences (rings) from of *T. elongatus* (PDB ID: 1JB0) [6,91], *A. marina* (PDB ID: 7COY) [39], *H. hongdechloris* (PDB ID: 6KMX) [56] and *F. thermalis* (PDB ID: 7LX0) [57]. Conserved residues are shown in cyan, and non-conserved residues are especially noted in the P700-cofactor environment. Numbering of residues is based on the *T. elongatus* sequence for consistency across species. For example, Thr743A (*T. elongatus*) is Ser in *H. hongdechloris*/*A. marina*, and Ala658A (*T. elongatus*) is Ser in *F. thermalis*. These differences lie adjacent to water-mediated H-bond networks and are expected to influence the hydrogen bonding and electrostatic stabilization of the P700 chlorophylls. The alignment highlights how specific substitutions in far-red–adapted species may tune the PSI protein–cofactor interface.

To evaluate sequence conservation within the local protein environments surrounding the primary donor branches, residues located within a 15 Å radius of the central Mg atom of the reaction center chlorophylls in both the A and B-branches were selected. This distance ensures inclusion of all amino acids potentially contributing to electron transfer. Residues within this cutoff were first identified from the *T. elongatus* structure and subsequently used as a reference for cross-species alignment. Conservation was assessed by comparing the total number of residues per branch with the number of identical residues at corresponding positions in *A. marina*, *H. hongdechloris*, and *F. thermalis*. The percentage of conservation was then calculated as the ratio of identical to total residues. Based on this analysis, *A. marina* exhibited 79% conservation in the A-branch and 68% in the B-branch, *H. hongdechloris* showed 79% conservation in both branches, and *F. thermalis* displayed 80% and 77% conservation in the A and B-branches, respectively, (Figure 4). Nevertheless, several amino acids surrounding the primary donor in PSI from *T. elongatus*, *A. marina*, *H. hongdechloris* and *F. thermalis* display significant variations. Notably, in the vicinity of P_B_, Tyr727 in *F. thermalis* is replaced by Thr743 in *T. elongatus*. Conversely, in the case of P_A_ Thr776 in *F. thermalis* is substituted by Tyr727 in *T. elongatus*. Additionally, for P_A_ Ala658 in *T. elongatus* corresponds with Ser635 in *F. thermalis*, while Val661 in *T. elongatus* corresponds to Leu638 in *F. thermalis*. For P_B_, Val694 in *F. thermalis* aligns with Leu637 in *T. elongatus*. These critical differences could highlight the variability of amino acids surrounding the primary donor between these two species. Further investigations and an expanded analysis of the protein environment could reveal insightful similarities and differences between the two organisms.

### 3.5. Comparison of Water Molecules

A comprehensive comparative analysis of the protein environment of *T. elongatus* and *F. thermalis* revealed notable differences, especially in the number and location of water molecules and putative H bonding interactions. A significant distinction is the lack of water molecules in *F. thermalis*. This absence suggests a possible adaption or structural feature that differentiates *F. thermalis* from *H. hongdechloris* and *T. elongatus*, despite general similarities in the protein environments. Analyses utilizing high-resolution structural data (PDB ID: 6PNJ and 7LX0) [57,81] disclose nuanced yet significant variations in the orientation of critical amino acid side chains in the vicinity of the primary donor, P_700_ and P_740_. The variations, frequently within 1 Å, influence the capacity of the Chls to interact with water molecules or neighboring amino acids.

It appears that the lack of water molecules in *F. thermalis* impairs the formation of the putative H bonding networks that are observed in *T. elongatus*. The Tyr727 residue in *F. thermalis* correlates to Thr743 in *T. elongatus*, with analogous shifts noted for other residues, such as, Ala658 in *T. elongatus* and Ser635 in *F. thermalis*. Additional computational modeling and experimental validation are required to better understand the effects of the structural changes on the stability and efficiency of electron transfer. This could yield insights on the evolutionary adaptation of cyanobacteria to different wavelengths of light and may reveal novel opportunities for bioengineering applications.

## 4. Conclusions

This review provides an overview of the structural differences in PSI of several species of cyanobacteria—specifically *A. marina*, *H. hongdechloris*, and *F. thermalis*— which have evolved to use different wavelengths of light and in the case of *H. hongdechloris* acclimate to far-red light conditions. Perhaps, somewhat surprisingly, the structural differences between PSI from these three species are rather minor. The most significant is the use of pheophytin as the eC-A3 acceptor in *A. marina*, which appears to have occurred to maintain the necessary driving force for forward electron transfer when the absorption wavelength of the donor is lowered. However, there are also subtle differences in the hydrogen bonding networks and the arrangement of water molecules around the primary donors, which may have arisen to fine tune their redox properties.

Interest in the far-red light adaptation of cyanobacteria is driven in part by the possibility of improving crop yields and land use by increasing photosynthetic efficiency at long wavelengths. The fact that cyanobacteria have been able to adapt to low-intensity, long-wavelength light environments, with relatively minor adjustments to their photosynthetic apparatus, suggests that it may be possible to engineer similar traits in higher plants. This could lead to the development of bioengineered crops and algae that can thrive in a wider range of environmental conditions, potentially increasing agricultural productivity and sustainability and addressing global challenges in food security and sustainable energy production.

## Data Availability

The original contributions presented in this study are included in the article. Further inquiries can be directed to the corresponding author.
